# Prediction of Three-Dimensional Soft Tissue Changes Following Mandibular PEEK Implantation (Proof of Concept)

**DOI:** 10.3390/jpm16080427

**Published:** 2026-08-13

**Authors:** Tom L. Zwijnenberg, Rutger H. Schepers, Johan Jansma, Haye H. Glas

**Affiliations:** 1 Clinic for Virtual Surgical Planning, 3D VSP B.V., Hoogwatum 2, 9906 TD Bierum, Groningen, The Netherlands; 2 Department of Oral and Maxillofacial Surgery, Expertcenter for Orthofacial Surgery, Martini Hospital Groningen, Van Swietenplein 1, 9728 NT Groningen, Groningen, The Netherlands

**Keywords:** PEEK implants, soft tissue prediction, mass tensor model, craniofacial surgery, mandibular reconstruction, computational modelling

## Abstract

**Background/Objectives**: Polyetheretherketone (PEEK) implants are increasingly being used for mandibular reconstruction and augmentation, yet predicting the resulting facial soft tissue changes remains challenging. Accurate pre-operative prediction of soft tissue deformation is essential for surgical planning and patient counselling. To the best of our knowledge, this study presents the first application of a computational model for predicting three-dimensional facial soft tissue deformation induced by PEEK implants. **Methods**: Four patient cases with pre-operative and post-operative cone-beam computed tomography scans were analysed. A mass tensor model was employed to predict soft tissue changes based on the pre-operative soft tissue shape, mandibular anatomy, and the designed PEEK implant. Model accuracy was quantified by comparing predicted outcomes to post-operative scans using landmark-based analysis of chin and jaw positions, as well as surface distance mapping, with a mean error below 2 mm adopted as the criterion for clinically acceptable accuracy. **Results**: Landmark-based analysis revealed a mean absolute error of 0.9 ± 0.8 mm between the predicted and actual positions. The mean surface distances were 1.3 mm for the jaw–chin region and 1.0 mm for the entire facial region. **Conclusions**: These results demonstrate the potential of a computational approach for real-time prediction of facial soft tissue changes following PEEK implant placement, achieving clinically relevant accuracy at the cohort level, with a tendency towards under-prediction and one case exceeding the threshold.

## 1. Introduction

Defects of the craniofacial complex can result in functional impairment, aesthetic disharmony, disfigurement and psychological distress [1]. The mandible and chin play a central role in facial symmetry and contour, and their morphology strongly influences perceived attractiveness and harmony [2,3]. Established surgical techniques, such as bilateral sagittal split osteotomy (BSSO) and genioplasty, enable the repositioning of the mandible and chin; however, alloplastic augmentation has gained interest since it provides greater freedom in contour design and adds volumetric support to the overlying soft tissue while avoiding osteotomy limitations [4].

Mandibular contour augmentation can be achieved with autologous bone or fat grafts, or with alloplastic materials such as silicone, porous polyethylene, titanium, and polyetheretherketone (PEEK), which differ in dimensional stability, ease of revision, and radiological behaviour [4,5]. Among these, PEEK is increasingly being used in craniofacial surgery due to its biocompatibility, radiolucency, and favourable elastic modulus [4,6,7]. With the advances in three-dimensional virtual surgical planning (3D VSP), patient-specific implant placement can be planned with sub-millimetre geometric precision [8,9,10]. Nevertheless, accurately predicting the overlying soft tissue shape remains considerably more challenging. This limitation is particularly relevant in aesthetic mandibular augmentation, where the primary surgical goal is to improve the external appearance, and the implant design cannot be altered intra-operatively. The absence of a validated soft tissue prediction tool represents a critical gap in the current PEEK planning workflow.

Although no soft tissue prediction model has been developed yet for PEEK implants, substantial groundwork exists in the field of orthognathic surgery. A notable shift has occurred from empirical two-dimensional soft-to-hard tissue ratio predictions to three-dimensional computational models [11]. Several modelling frameworks have been explored for this purpose, including finite element models [12,13,14,15,16], mass-spring models [16,17,18], mass tensor models [16,19,20,21,22,23] and deep-learning approaches [24,25,26,27]. These frameworks differ in their underlying assumptions and applicability to PEEK implant scenarios, but offer a valuable foundation from which a dedicated prediction model can be developed and benchmarked. Among them, mass tensor models have emerged as a hybrid solution that uses pre-computed stiffness tensors to enable fast, clinically viable predictions without requiring a large training dataset.

To our knowledge, no study has evaluated the accuracy of any computational model for predicting three-dimensional soft tissue deformation, specifically following PEEK implant placement in the mandibular region. This proof-of-concept study therefore aimed to quantify the agreement between the predicted soft tissue by a real-time mass tensor model and the actual post-operative morphology, using the post-operative CBCT-derived soft tissue surface as the reference standard. Accuracy was expressed as the Euclidean distance between the predicted and post-operative surfaces, both at relevant landmarks (Pogonion and Gonion) and as surface-to-surface distances over the jaw–chin region and over the entire face. Mean prediction errors of approximately 0.3 to 2.9 mm have been reported for comparable models in orthognathic surgery [16,19,20,21,22,23], and deviations below 2 mm are generally regarded as clinically imperceptible [18,24,28,29,30]. A mean error below 2 mm was therefore adopted as the criterion for clinically acceptable accuracy in this study.

## 2. Materials and Methods

### 2.1. Study Design and Patient Selection

This proof-of-concept study included a consecutive cohort of four patients who underwent mandibular augmentation surgery, using patient-specific PEEK implants, at the Martini Hospital (Groningen, The Netherlands) between November 2023 and October 2025. The study protocol was reviewed by the Medical Ethics Committee of the Martini Hospital Groningen, which determined that the study is exempt from review under the WMO (approval number 2025-006, 3 March 2025). Patients were selected based on the criteria outlined in Table 1.

### 2.2. Data Acquisition

Pre-operative and post-operative cone-beam computed tomography (CBCT) scans were acquired for each patient. Post-operative imaging was performed approximately 10 weeks after surgery to allow for resolution of the majority of acute post-operative swelling. Hard and soft tissue segmentation of all the CBCT datasets was performed using a routine automated workflow and visually inspected by an experienced technical physician using Materialise Mimics (Mimics Medical 27.0; Materialise NV, Leuven, Belgium). An identical protocol was applied to the pre- and post-operative datasets of each patient. The segmented structures served as the basis for both the implant design and subsequent prediction.

### 2.3. Virtual Surgical Planning and Implant Fabrication

A three-dimensional virtual surgical planning (3D VSP) digital workflow was performed for each patient by an experienced maxillofacial surgeon and technical physician. The resulting three-dimensional models were imported for implant development into Materialise 3-Matic (3-Matic Medical 18.0; Materialise NV, Leuven, Belgium). Patient-specific implants (PSIs) were designed to achieve the patient’s individual aesthetic objectives, including goals such as increased chin projection, enhanced mandibular angle definition, or a more harmonious lower facial contour.

**Figure 1:**
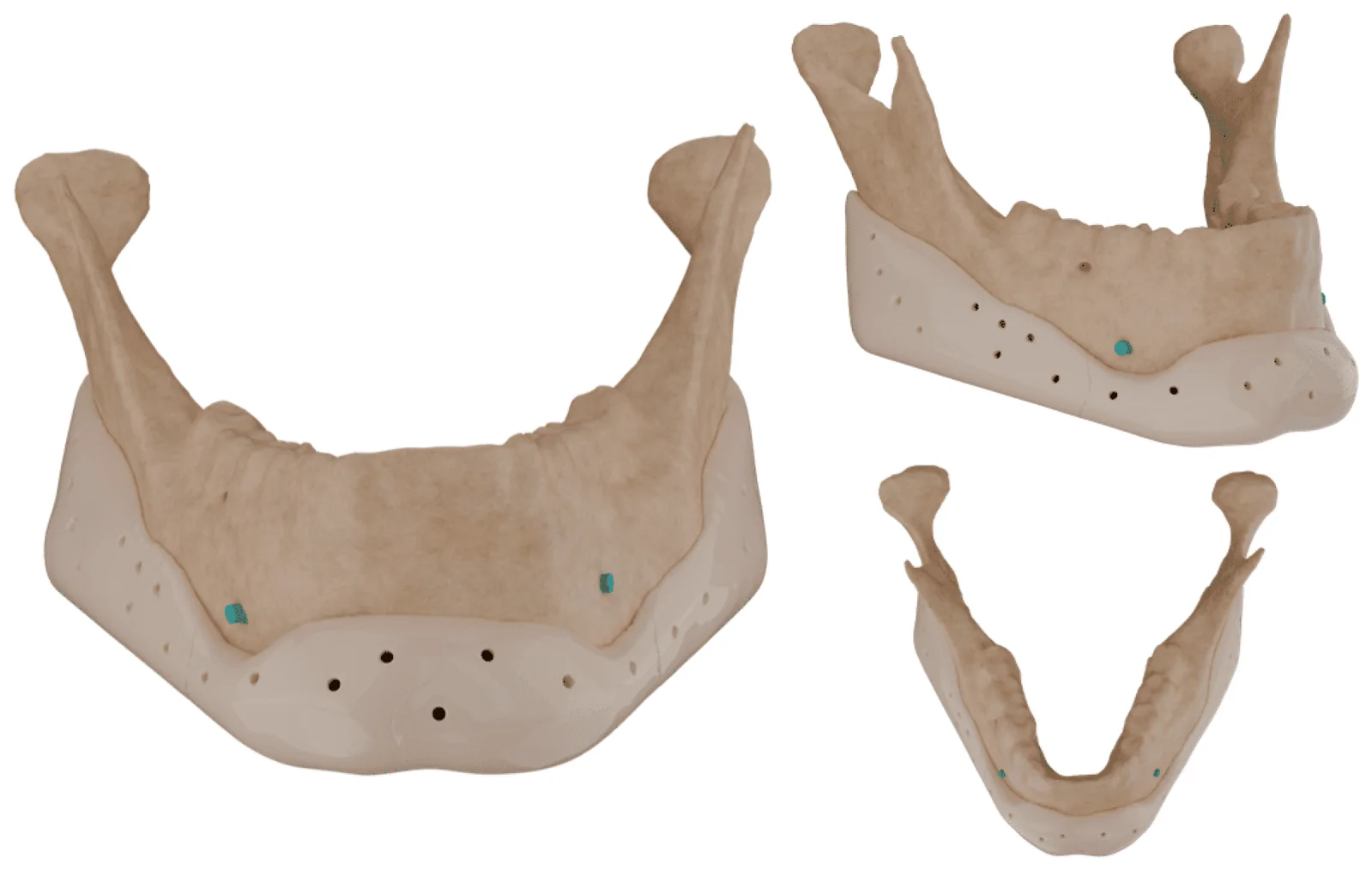
Top, ventral and side view of a patient-specific PEEK implant shown together with the corresponding mandibular bone. The inferior alveolar nerve is rendered in blue, illustrating its preserved spatial distance from the implant. Multiple pre-drilled screw holes allow for intra-operative selection of fixation sites.

The final implant designs were manufactured from PEEK by 3D VSP (3D VSP B.V., Bierum, The Netherlands) using a Kumovis 3D printing machine. To accommodate intra-operative positioning flexibility, each implant segment was manufactured with multiple pre-drilled screw holes corresponding to the planned fixation sites, as shown in Figure 1.

### 2.4. Surgical Procedure

Vestibular incisions were made to provide subperiosteal access to the mandibular bony surface. The implants were seated directly onto the cortical bone, using their bone-borne interface geometry to achieve precise passive positioning. Fixation was achieved using 2.0 mm MaxDrive titanium screws (KLS Martin, Tuttlingen, Germany), with the number and position of the fixation points being determined intra-operatively.

### 2.5. Soft Tissue Prediction Workflow

**Figure 2:**
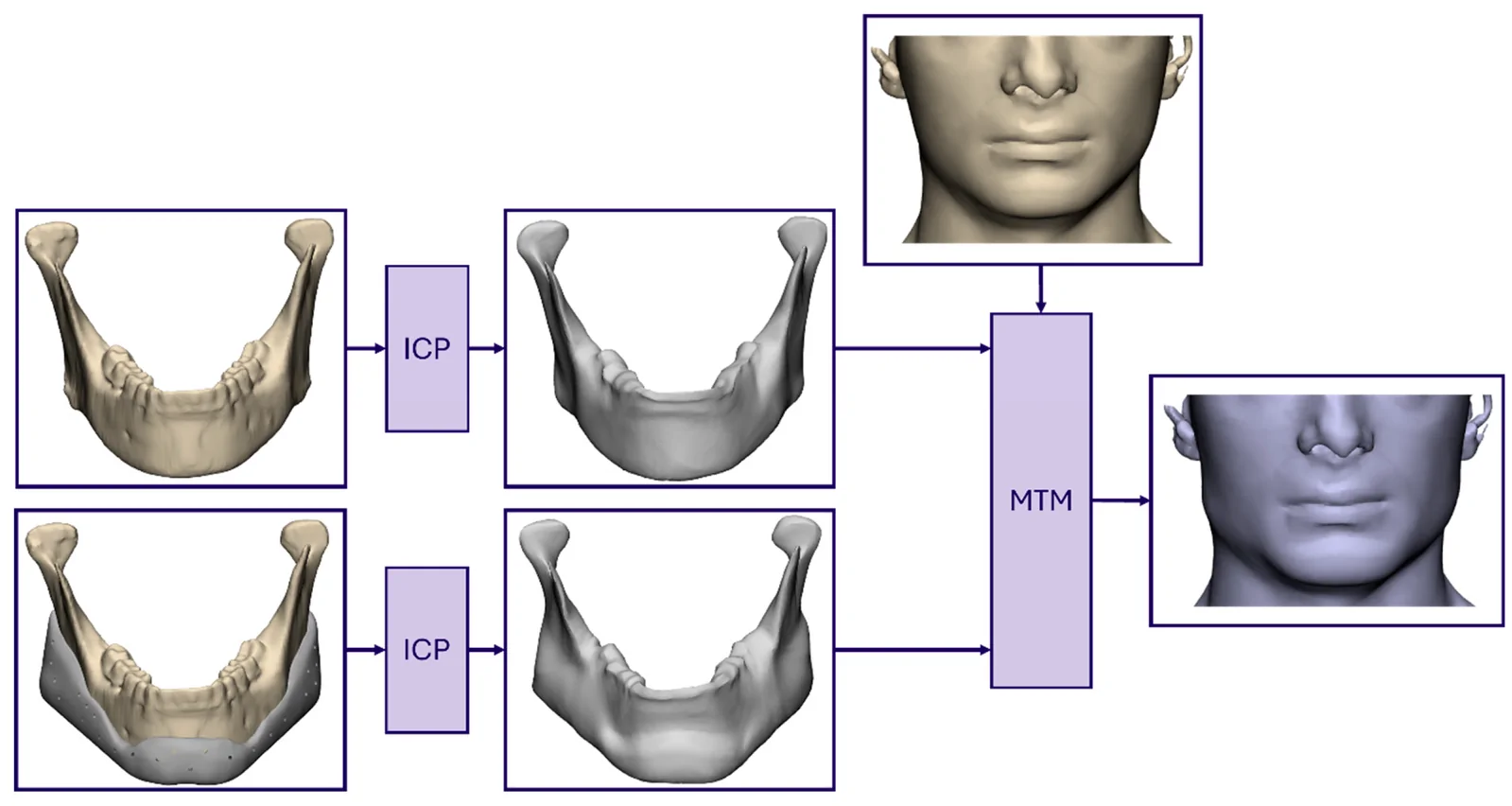
Overview of the multi-step workflow for predicting facial soft tissue changes. A template mandibular mesh is registered to both the pre-operative and augmented (native bone plus PEEK implant) mandibular surfaces using a non-rigid iterative closest point (ICP) algorithm to establish vertex correspondence. The mass tensor model (MTM) is then initialised on the pre-operative soft tissue surface and applies the hard tissue displacement as a boundary condition to predict the post-operative soft tissue deformation.

Three-dimensional soft tissue deformation was predicted using a mass tensor model (MTM) implemented in Python (version 3.14.4; Python Software Foundation, Wilmington, DE, USA) (Supplementary Algorithm S1). The prediction followed a standardised multi-step workflow, illustrated in Figure 2 and described in the following subsections.

Hard Tissue Correspondence. A template mandible mesh was registered to both the pre-operative and post-operative mandibular shapes (native bone plus PEEK implant) using a non-rigid iterative closest point (ICP) algorithm. This method of integrating morphable mandibular shape representations allows for vertex-to-vertex correspondence between the native and augmented jaw shape.

Mesh Standardisation. The pre-operative soft tissue mesh was resampled to ensure a uniform distribution of vertices across the surface, important for prediction stability and ensuring consistency across different patient geometries.

Displacement Calculation. Displacement vectors were computed between corresponding vertices on the pre-operative bone surface and the designed PEEK implant surface. These vectors represented the local hard tissue deformation at each point and were used as boundary conditions for the subsequent soft tissue prediction.

MTM Application. The mass tensor model implemented in this study, shown in Figure 2, was based on the framework described by Mollemans et al. [16]. The mass tensor for each skin surface node was initialised as the identity matrix $M_{i}=I_{3\times 3}$, representing homogeneous elastic behaviour. The influence of any mandibular displacement on each soft tissue vertex was determined using a Gaussian distance-based weighting scheme. The weights were computed from the Euclidean distances between the soft tissue and bone vertices using a k-nearest neighbour search:

(1) $$\begin{array}{c} \omega_{ij}=\frac{\exp\;(-\frac{d_{ij}^{2}}{2\sigma^{2}})}{\;\sum_{k=1}^{K}\exp\;(-\frac{d_{ik}^{2}}{2\sigma^{2}})}, \end{array}$$

where $d_{ij}$ is the Euclidean distance between the soft tissue vertex i and the mandibular vertex j, K is the number of the considered nearest bone vertices, and σ the Gaussian bandwidth. In this study, the parameters were empirically set to K=300 and σ=2. The resulting surface node displacement vector was defined as follows:

(2) $$u_{i}=\sum_{j=1}^{K}\omega_{ij}d_{j},$$

where $d_{j}$ denotes the displacement vector of the mandibular vertex j. Nearest-neighbour searches were performed by implementing a k-d tree to enable efficient spatial queries.

### 2.6. Accuracy Evaluation

The post-operative CBCT-derived soft tissue surface served as the reference standard against which the predicted surface was compared. Both the pre- and post-operative soft tissue segmentations were co-registered based on the unaltered cranial skeleton. Accuracy was assessed using two complementary methodologies.

#### 2.6.1. Landmark-Based Analysis

**Figure 3:**
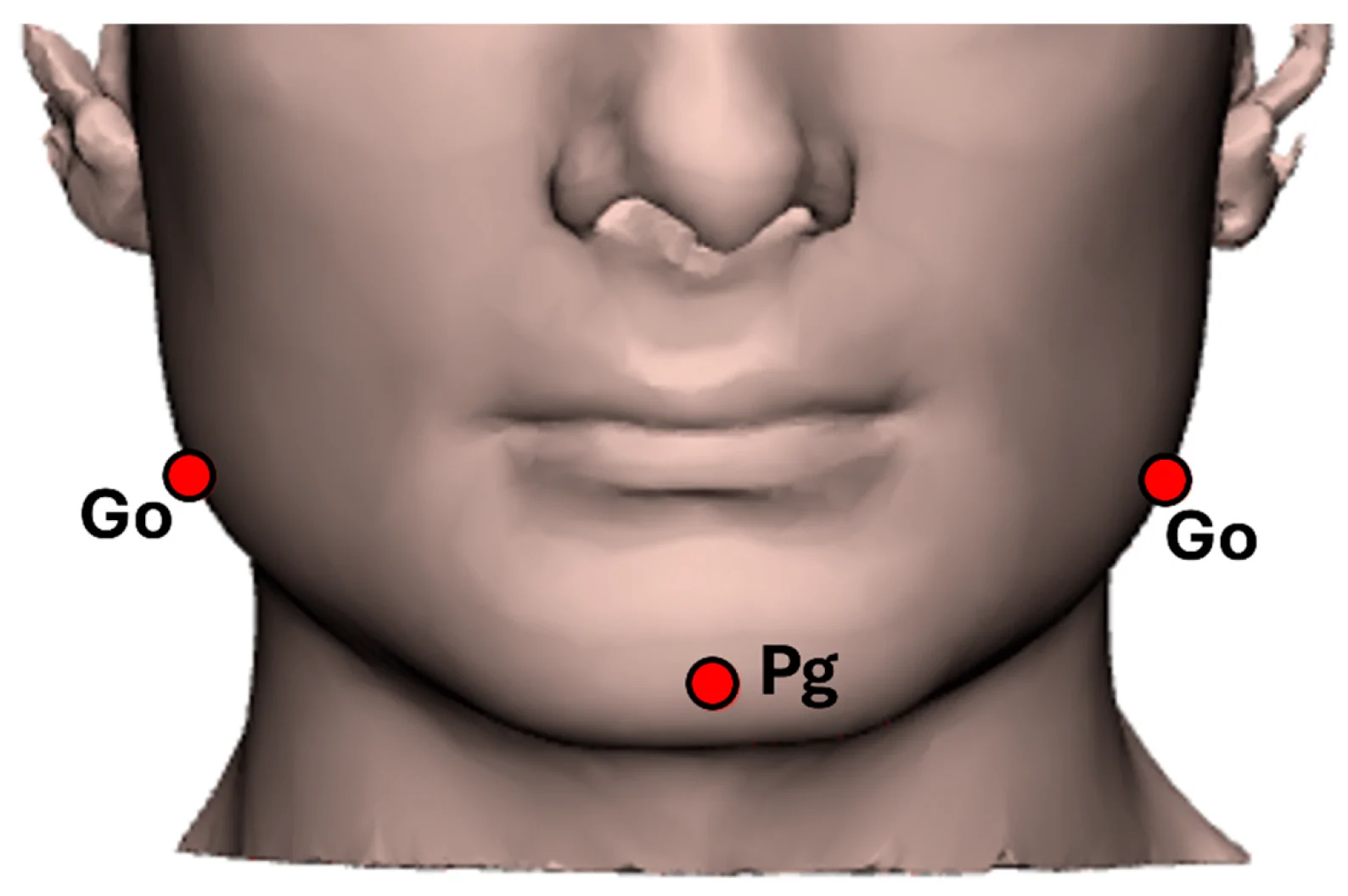
Three-dimensional visualisation of the facial soft tissue surface showing the anatomical landmarks used for accuracy evaluation. The Pogonion (Pg) landmark is positioned on the chin, and the bilateral Gonion (Go) landmarks are positioned at the mandibular angles.

Soft tissue landmarks were derived semi-automatically from the corresponding hard tissue landmarks. All scans were aligned to the natural head position of the patient using two-dimensional photographs, which defined the coordinate frame used throughout this study. On the mandibular surface, the bony Pogonion was defined as the most anterior point and the bony Gonion as the most inferior point of the mandibular angle on each side. The corresponding soft tissue landmark, shown in Figure 3, was obtained by projecting each bony landmark along a fixed anatomical axis until it intersected the soft tissue surface: in the antero-posterior direction for the Pogonion (Pg) and in the medio-lateral direction for the left and right Gonion (Go). The projection ray originated from the pre-operative bony landmark and was applied identically to the pre-operative, predicted and post-operative soft tissue surfaces.

#### 2.6.2. Surface Distance Mapping

A three-dimensional surface-to-surface distance map was generated between the predicted and actual post-operative soft tissue meshes. Surface-to-surface distances were computed separately for two regions. The jaw–chin region was defined as the soft tissue area directly overlying the implant, selected individually for each patient. Additionally, the entire facial surface was evaluated to assess global prediction stability and registration quality.

### 2.7. Statistical Analysis

Given the proof-of-concept design and the limited number of included cases, the analysis was descriptive, and no statistical testing was performed. Landmark error was expressed as the signed distance between the predicted and post-operative landmark positions. Surface accuracy was expressed as the mean surface-to-surface distance over the jaw–chin region and over the entire facial surface. All metrics are reported as mean ± standard deviation at the cohort level, with per-case values presented separately, alongside the root mean squared error and the percentage within 2 mm. It should be noted that the landmarks within a single patient are not statistically independent, which further precludes formal hypothesis testing in a cohort of this size.

### 2.8. Sensitivity Analysis

To assess the robustness of the model to the choice of parameters, a sensitivity analysis was performed in which each parameter was varied in turn while the remaining parameters were held at their reference values (K = 300, σ = 2). The number of contributing mandibular vertices K was varied from 25 to 1000 and the bandwidth scale factor σ from 0.25 to 12. For each setting, the prediction was recomputed and the mean surface distance to the post-operative surface was evaluated over a fixed evaluation region, held identical across all settings so that comparisons were like-for-like. The analysis was performed for all four cases.

## 3. Results

**Figure 4:**
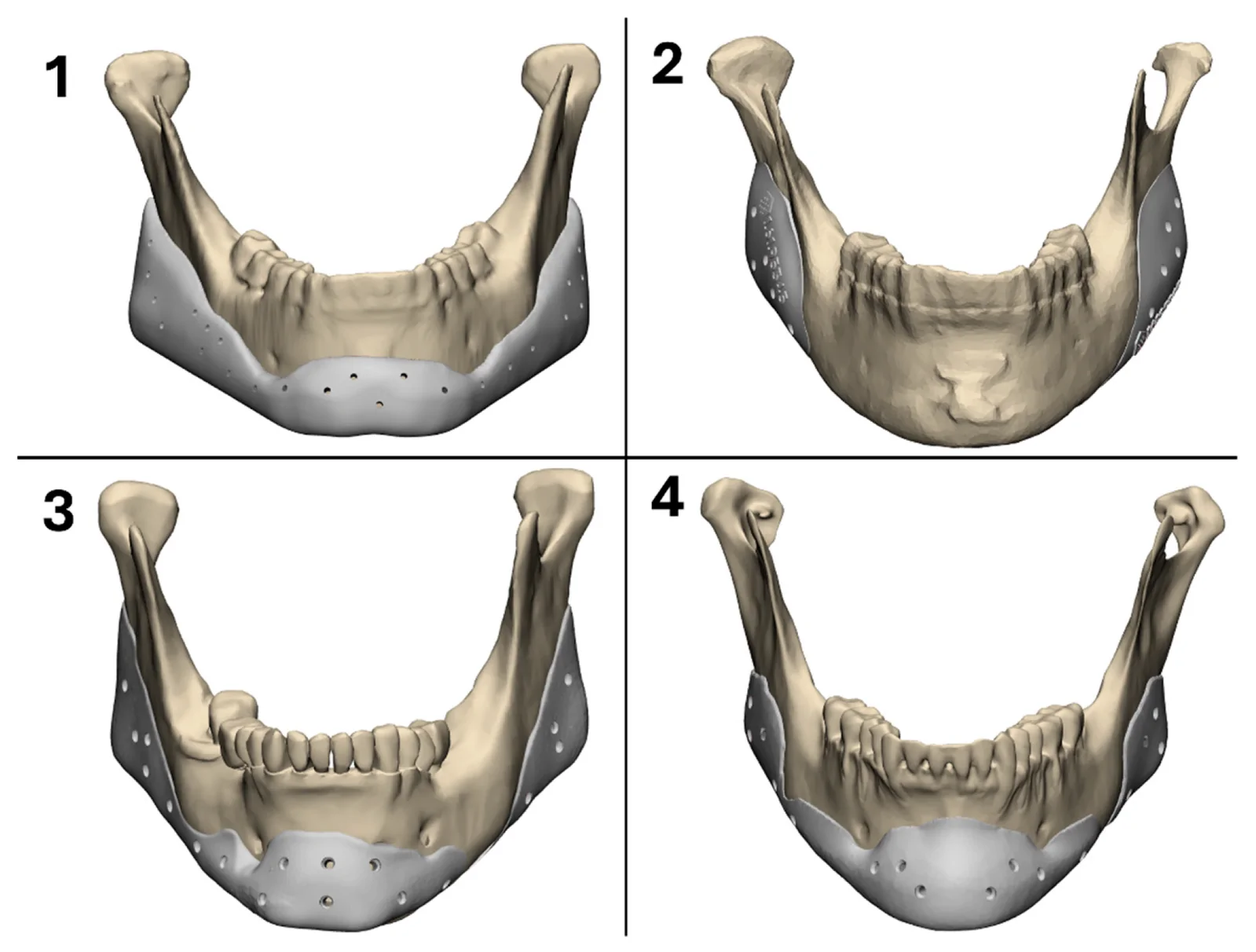
The designed patient-specific PEEK implants for the four included cases. Cases (**1, 3**, and **4**) received a mandibular wrap-around implant, whereas Case (**2**) received bilateral implants restricted to the gonial regions.

The mass tensor model was applied and evaluated in four patients undergoing mandibular augmentation with patient-specific PEEK implants. The original mandibular shapes with the designed PEEK implants are illustrated in Figure 4. Three patients received a mandible wrap-around implant, while one patient (Case 2) received implants restricted to the bilateral gonial region; hence, the Pogonion landmark was excluded from the analysis.

Landmark-based analysis was performed on eleven landmarks across the four cases: the bilateral Gonion in all patients and the Pogonion in the three patients who received a wrap-around implant (Table 2). The planned implant width at the landmarks ranged from 3.9 to 11.4 mm (mean 6.8 ± 2.3 mm), with a corresponding mean predicted soft tissue change of 3.5 ± 1.7 mm. The mean error between the predicted and post-operative landmark positions was −0.4 ± 1.2 mm, while the mean absolute error was 0.9 ± 0.8 mm. The signed errors were negative at eight of the eleven landmarks, indicating a tendency of the model to predict slightly less soft tissue change than was observed. This tendency was largely attributable to Case 3; excluding this case, the mean signed error was 0.0 mm across the remaining eight landmarks.

**Figure 5:**
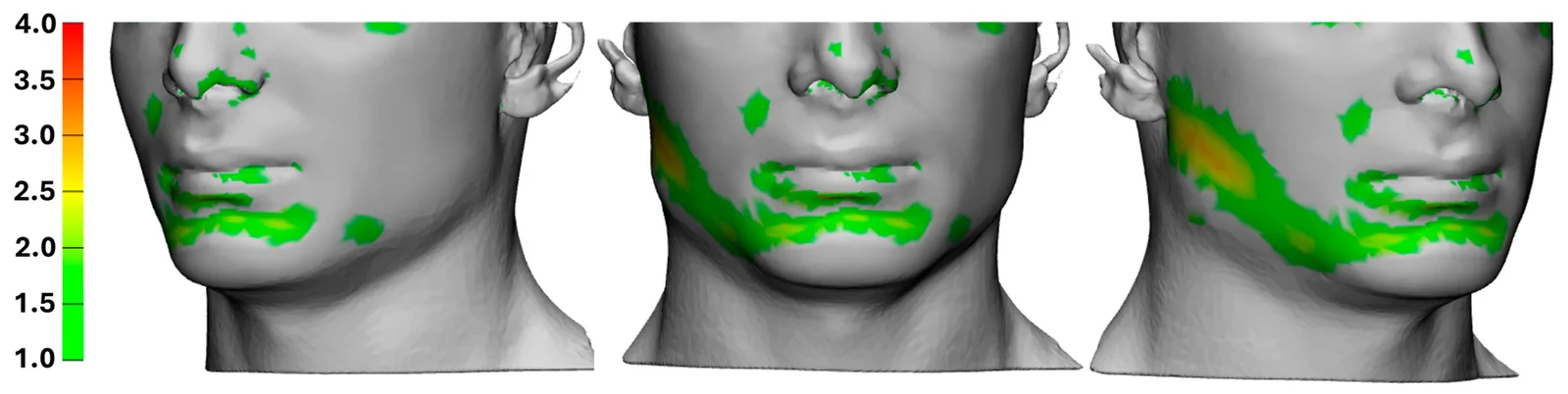
Surface distance map between the predicted and post-operative soft tissue surfaces across the entire facial region for Case 1. The colour scale represents the absolute Euclidean distance (in millimetres) between the two surfaces, with warmer colours indicating larger deviations. Distances below 1 mm are shown in grey.

The quantitative surface distance results are presented in Table 3. All cohort mean values remained below the 2 mm threshold. The surface distance between the predicted and post-operative soft tissue surfaces across the entire facial region is visualised for a representative case in Figure 5. At the individual level, only the jaw–chin surface error of Case 3 exceeded this threshold (2.1 mm). Surface distance mapping of the jaw–chin region yielded a mean error of 1.3 mm, while mapping of the entire facial surface resulted in a mean error of 1.0 mm. The smallest jaw–chin surface error was observed in Case 2 (0.9 mm), and the smallest entire face error was observed for Cases 1 and 4, both at 0.7 mm.

The mean distance between the pre- and post-operative surfaces, representing the soft tissue change actually produced by the procedure, was 2.2 mm over the jaw–chin region and 1.2 mm over the entire face. Relative to this baseline, the prediction reduced the mean surface distance to 1.3 mm and 1.0 mm, respectively, corresponding to a reduction of 38% over the jaw–chin region and 18% over the entire face. The proportion of the region predicted within 2 mm was 76.0% for the jaw–chin region and 87.2% for the entire face.

**Figure 6:**
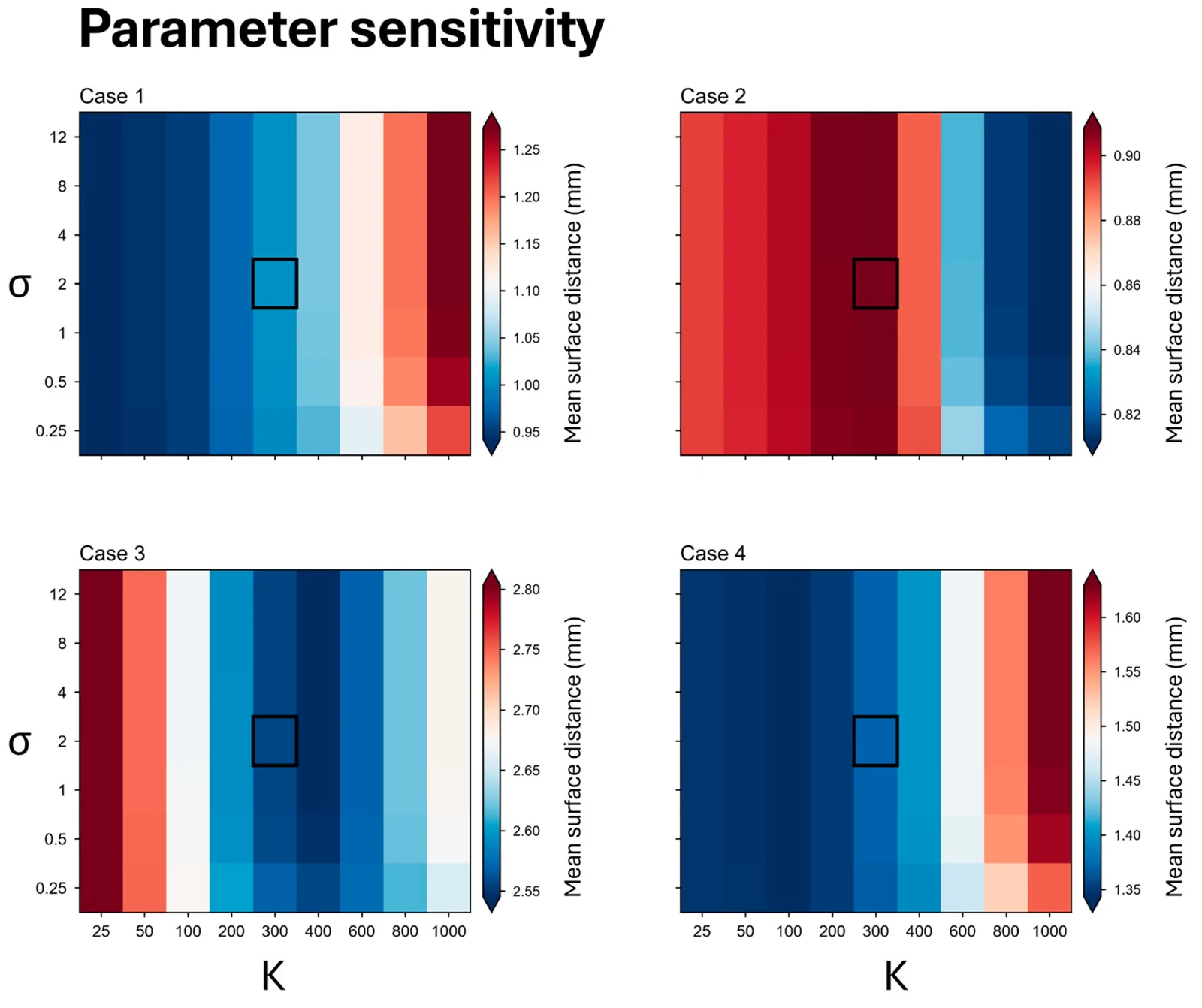
Each panel shows one case. Cell colour encodes the mean absolute surface distance (mm) between the predicted soft tissue surface and the post-operative jaw–chin region, for every combination of K on the *x*-axis and σ on the *y*-axis. The black rectangle marks the default settings used for the predictions in this study (K = 300, σ = 2). Each panel carries its own colour scale and colour bar.

The results of the sensitivity analysis are shown in Figure 6. Across the 63 parameter combinations tested per case, the response surface was flat in the vicinity of its minimum. The default setting used throughout this study lay within this broad minimum in every case. Adopting this single global setting rather than the per-case optimum increased the mean surface distance by 0.01 to 0.10 mm, corresponding to 0.4% to 11.9% of the per-case optimal error.

**Figure 7:**
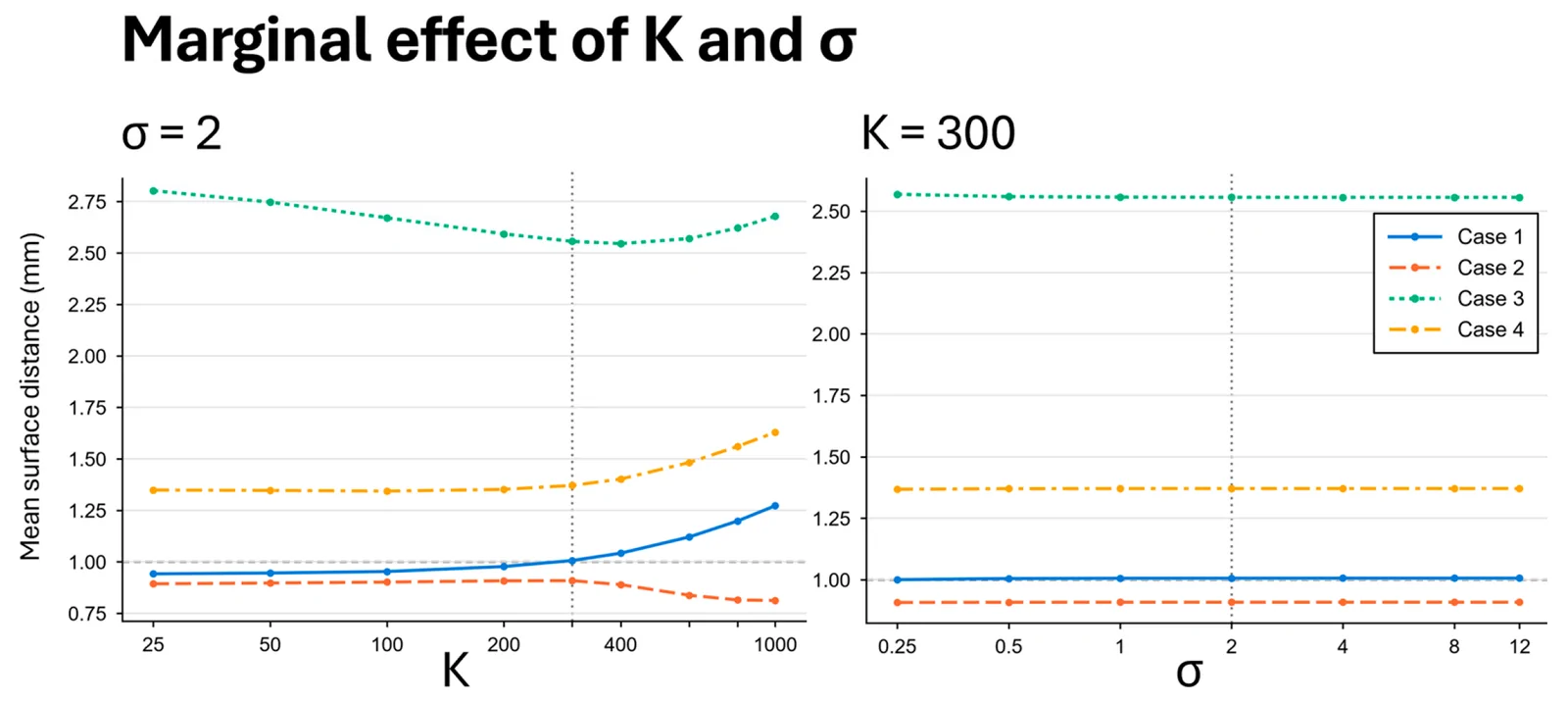
Mean absolute surface distance to the post-operative jaw–chin region as a function of K at fixed σ = 2 (**left**) and as a function of σ at fixed K = 300 (**right**). Both panels share the *y*-axis, so the two are directly comparable. The dotted vertical line marks the default value of the parameters used for the predictions in this study.

The two parameters differed in their influence, also shown in Figure 7. Averaged over the grid, varying K changed the mean surface distance by 0.10 to 0.32 mm depending on the case, whereas varying σ changed it by <0.01 to 0.02 mm. The influence of K was considerably larger, and its per-case optima did not converge on a common value. They occurred at K = 25, 1000, 400 and 100 for Cases 1 to 4, respectively, spanning the entire tested range.

## 4. Discussion

This study presents the first application of a soft tissue prediction model for mandible-enhancing PEEK implants. Using a mass tensor model that integrates morphable mandibular shape representations, real-time soft tissue predictions were generated for four clinical cases. Across the cohort, the model achieved a mean surface distance error of 1.3 mm in the jaw–chin region, 1.0 mm across the entire facial region, and a mean absolute landmark accuracy of 0.9 mm. Evaluation showed that all these accuracy values remained within the 2 mm clinical relevance threshold [18,24,28,29,30].

The existing body of evidence on three-dimensional soft tissue prediction has been largely derived from orthognathic surgery, where skeletal movements are the primary driver of soft tissue change. In that context, the reported accuracies range from approximately 0.3 mm to 2.9 mm depending on the modelling approach and evaluation methodology [16,19,20,21,22,23].

The landmark-based and surface-to-surface accuracies achieved in the present study are consistent with the range reported for MTM-based approaches used in orthognathic surgery, with typical landmark distances between 0.3 and 2.5 mm [16,19,20,21,22,23]. This agreement is notable given that PEEK implant augmentation introduces a distinct mechanical challenge: rather than relocating existing bone, the implant adds volume beneath the soft tissue envelope, inducing a predominantly outward stretching deformation that differs biomechanically from translational or rotational skeletal movements. These results support the feasibility of the mass tensor approach, although the small number of cases precludes any firm conclusion.

Interpreting the surface metrics requires a reference point. Since the prediction error is expressed as a distance to the post-operative surface, it should be judged against the distance obtained by predicting no change at all. Over the jaw–chin region the procedure produced a mean change of 2.2 mm and the model reduced the residual distance to 1.3 mm, a reduction of 38%. Over the entire face, the corresponding reduction was 18%. The whole face figure should not be read as a measure of predictive performance. Most of the surface is not displaced by the implant, so the metric is dominated by residual disagreement. It therefore functions primarily as a check on registration and segmentation consistency. The jaw–chin error of 1.3 mm sits above this floor by a margin that is real but modest in a cohort of four, and the proportion of that region predicted to within 2 mm (76.0%) conveys the same result in a form that is more directly interpretable for implant design.

The higher standard deviation observed for the jaw–chin region compared with the entire facial surface reflects greater variability in the prediction in the area directly overlying the implant, where soft tissue change actually occurs and the model is most challenged. By contrast, the regions of the face away from the implant footprint are expected to remain largely unchanged between the pre- and post-operative scans. In Cases 1 and 4, the entire face surface errors were both 0.7 mm, indicating that the predicted displacement propagated correctly into the surrounding soft tissue. At the landmark level, the signed errors were negative at eight of the eleven landmarks, with a mean of −0.4 ± 1.2 mm, indicating that the model tended to under-predict the magnitude of soft tissue change. This tendency was driven largely by Case 3, in which the removal of a pre-existing silicone implant was performed in the same procedure. Although the geometry of the pre-existing implant was represented in the model, soft tissue that has adapted to a long-standing alloplastic implant may respond differently to renewed augmentation than unoperated tissue. Changes in the overlying tissue are not captured by the homogeneous elastic assumptions of the model and provide a plausible explanation for the reduced prediction accuracy in the implant-overlying region for this case. With this case excluded, the mean error across the remaining landmarks is 0.0 mm. Also, residual post-operative oedema at the ten-week imaging interval could increase the observed soft tissue change and bias the signed error in the negative direction [31,32].

The sensitivity analysis showed that the prediction is governed almost entirely by K, the number of contributing mandibular vertices. This follows from the structure of Equation 1. Because the weights are normalised over the K selected neighbours, σ redistributes weight within a fixed neighbourhood rather than altering its spatial extent. K determines how far the influence of the hard tissue displacement reaches and therefore whether vertices carrying dissimilar displacements enter the average.

The response surface was flat in the vicinity of its minimum in all four cases, and the default setting (K = 300, σ = 2) fell within this region. The prediction is therefore robust to the parameter choice within the range that matters clinically, which supports the use of a single fixed setting across patients. The per-case optima did not converge, however, occurring at K = 25, 1000, 400 and 100 and spanning the entire tested range. Whether the influence radius should be adapted per patient, for instance to local soft tissue thickness, remains open and requires a larger cohort. It is worth noting that the accuracy reported here was not obtained by tuning K to the outcome: the parameters were fixed beforehand and lie away from the per-case optimum in all four cases.

Several external factors that are not captured in the current model may also contribute to residual prediction error. Post-operative swelling, gravitational effects, changes in muscular tension, and soft tissue ageing all influence the final soft tissue position [31,32,33,34]. The approximate ten-week interval between surgery and post-operative imaging was chosen to ensure that most of the acute swelling had resolved, thereby reducing but not eliminating the confounding effect of oedema.

An additional source of error inherent to the workflow is the segmentation accuracy of CBCT-derived soft tissue surfaces. CBCT achieves a soft tissue resolution of approximately 0.3 mm compared with the gold laser surface scanning standard [35]. Segmentation-related uncertainty therefore sets a practical lower boundary on the achievable validation accuracy, independent of the model’s intrinsic performance.

Several limitations should be considered in addition to those discussed above. First, the cohort comprised four patients and eleven landmarks. This is inherent to the proof-of-concept design, but it limits the precision of the reported accuracies and statistical analysis. Second, one patient had previously undergone augmentation of the chin which was replaced by PEEK during the procedure. Such cases were deliberately retained rather than excluded, as a substantial proportion of patients presenting for patient-specific PEEK augmentation have undergone prior alloplastic augmentation, and the model should ultimately be applicable to this group. Third, all patients were treated at a single centre by the same surgical team, and all segmentation and landmark identification were performed by a single observer, so neither inter-observer nor inter-centre variability could be assessed.

PEEK implants increase the mandibular volume, leading to stretching of the soft tissue surface. Studies show that soft tissue in the chin area contains different elastic properties than the cheeks [36,37]. In the genioplasty literature, a ratio of approximately 0.9:1 has been established [38], whereas the data used in this study showed a mean ratio of 0.7:1 at the Pogonion following PEEK augmentation. Whether this difference reflects a genuine biomechanical distinction between augmentation and osteotomy, or is attributable to the small sample size, requires confirmation with a larger dataset.

The clinical implications of a validated soft tissue prediction model are substantial for the PEEK augmentation workflow. Unlike orthognathic procedures, in which iterative skeletal planning and intra-operative adjustment remain feasible, patient-specific PEEK implants must be entirely designed and fabricated before surgery. The implant morphology is fixed at the time of manufacturing, making the pre-operative design phase the critical determinant of the aesthetic outcome. A reliable soft tissue prediction tool therefore enables the technical physician and surgeon to iteratively optimise the implant design against the expected soft tissue result, and to verify that the planned augmentation will achieve the patient’s individual aesthetic goals prior to fabrication.

Equally important is the role of such a model in pre-operative patient communication. In predominantly aesthetic procedures, clear and realistic visualisation of the expected post-operative appearance is fundamental to establishing informed consent and aligning patient expectations with the surgical outcomes. A mathematically grounded three-dimensional soft tissue prediction provides a more objective and individualised basis for this conversation than is currently available.

The present study can serve as a proof of concept and a benchmark for future model development. Several directions merit exploration. First, expanding the dataset to include a larger number of cases will improve the statistical power and allow for more robust characterisation of the model. Combining PEEK augmentation data with orthognathic surgery cases, for which larger datasets and established validation frameworks exist, could accelerate this process. Second, integrating deep learning with morphable mandibular shape representations employed in this study presents a promising direction. Several recent studies have demonstrated that deep-learning models can achieve high soft tissue prediction accuracy [24,25,26,27]. Combining their representational capacity with the morphable mandibular shape framework may yield further improvements.

## 5. Conclusions

This proof-of-concept study demonstrates the potential of a mass tensor model for predicting soft tissue changes following patient-specific PEEK mandibular augmentation. Across four cases, the model achieved a mean absolute landmark error of 0.9 ± 0.8 mm and mean surface distances of 1.3 mm over the jaw–chin region and 1.0 ± 0.4 mm over the entire facial surface, all of which remained below the 2 mm threshold of clinical relevance at the cohort level. Case 3 was the exception, exceeding this threshold both in the jaw–chin region (2.1 mm) and at a single landmark (−2.5 mm). The errors were negative at eight of the eleven landmarks, with a mean of −0.4 ± 1.2 mm, indicating a tendency of the model to under-predict the magnitude of soft tissue change. As the first application of such a model in this setting, these results establish a baseline and support the feasibility of integrating soft tissue prediction into the PEEK implant design workflow. However, confirmation in a larger cohort is required before clinical implementation. Future work should focus on expanding the dataset and exploring the integration of deep-learning approaches.

## Figures and Tables

**Table 1:** Patient inclusion and exclusion criteria.

Inclusion Criteria	Exclusion Criteria
PEEK implants for mandible wrap-around procedure or localised gonial augmentation	Unsuitable CBCT quality for analysis
Over 18 years of age with a fully matured maxillofacial complex	
Availability of both pre- and post-operative CBCT scans	

**Table 2:** Per-case landmark measurements at the Gonion and Pogonion. Implant width denotes the thickness of the PEEK implant; predicted change is the soft tissue displacement between the pre-operative and predicted surfaces; and error is the distance between the predicted and post-operative surfaces, with a positive value indicating that the predicted change exceeded the post-operative change. The Pogonion was excluded from Case 2, which received bilateral gonial implants only. All values are in millimetres; the bottom rows show the cohort mean ± standard deviation (SD) for the signed error and the mean absolute error.

	Landmark	Implant Width (mm)	Predicted Change (mm)	Actual Change (mm)	Error (mm)
Case 1	Gonion Left	9.9	6.2	6.0	0.2
	Pogonion	6.0	3.4	3.5	−0.1
	Gonion Right	6.5	5.5	5.8	−0.3
Case 2	Gonion Left	5.8	3.8	1.9	1.9
	Gonion Right	4.1	1.9	2.8	−0.9
Case 3	Gonion Left	11.4	3.2	5.7	−2.5
	Pogonion	6.3	1.4	3.2	−1.8
	Gonion Right	8.6	2.8	2.9	−0.1
Case 4	Gonion Left	3.9	1.8	2.2	−0.4
	Pogonion	7.5	6.1	7.2	−1.1
	Gonion Right	5.0	2.3	1.8	0.5
Mean ± SD		6.8 ± 2.3	3.5 ± 1.7	3.9 ± 1.9	−0.4 ± 1.2
MAE ± SD		—	—	—	0.9 ± 0.8
RMSE		—	—	—	1.2

Abbreviations: SD, Standard Deviation; MAE, Mean Absolute Error; RMSE, Root Mean Squared Error.

**Table 3:** Per-case surface-to-surface distances for the jaw–chin region and the entire facial surface. Baseline denotes the mean distance between the pre-operative and post-operative soft tissue surfaces, representing the magnitude of soft tissue change actually produced by the procedure. Prediction error represents the distance between the predicted and post-operative surfaces, reported as the mean ± standard deviation across all surface vertices within the region. Within 2 mm denotes the proportion of the region for which the prediction error was below the 2 mm threshold. The bottom row shows the cohort mean computed across the four cases. All distances are in millimetres.

	Jaw–chin Region			Entire Facial Surface		
Case (Mean ± SD)	Actual Change (mm)	Prediction Error (mm)	Within 2 mm (%)	Actual Change (mm)	Prediction Error (mm)	Within 2 mm (%)
Case 1	2.3 ± 1.3	0.9 ± 0.7	90.0	1.0 ± 1.0	0.7 ± 0.6	95.5
Case 2	1.2 ± 0.8	0.9 ± 0.7	91.8	1.1 ± 0.8	1.0 ± 0.7	90.2
Case 3	2.9 ± 1.5	2.1 ± 1.2	44.7	1.8 ± 1.5	1.6 ± 1.1	68.0
Case 4	2.2 ± 2.0	1.4 ± 1.1	77.6	1.0 ± 1.3	0.7 ± 0.7	95.2
Cohort Mean	2.2	1.3	76.0	1.2	1.0	87.2

Abbreviations: SD, Standard Deviation.

## Data Availability

Data are available on reasonable request to the corresponding author. The complete prediction algorithm, including all governing equations, parameter values and processing steps, is provided as pseudocode in Supplementary Algorithm S1. The underlying source code is not publicly available, as it forms part of the proprietary implant design workflow of 3D VSP B.V.

## Supplementary Materials

The following supporting information can be downloaded at: https://www.mdpi.com/article/10.3390/jpm16080427/s1, Algorithm S1. Pseudocode of the mass-tensor soft-tissue prediction workflow.
