# Supplementary material for: Prediction of Three-Dimensional Soft Tissue Changes Following Mandibular PEEK Implantation (Proof of Concept)
Source: J Pers Med. 2026 Aug 13;16(8):427. doi: 10.3390/jpm16080427 (PMC13514417; doi:10.3390/jpm16080427)
Supplement: Supplementary file 1 [file jpm-16-00427-s001.zip › jpm-4417658-supplementary.pdf]

Supplementary Material

Algorithm S1. Pseudocode of the mass-tensor soft-tissue prediction workflow

This supplement provides a complete algorithmic description of the soft-tissue prediction model used in this study, sufficient for independent reimplementation. The source code is not released, as it forms part of the proprietary implant design workflow of 3D VSP B.V. All governing equations, parameter values and processing steps are given below and correspond to Section 2.5 of the main manuscript.

1. Notation

Symbol	Definition	Units
`N`	Number of vertices of the pre-operative soft-tissue surface	-
`M`	Number of vertices of the mandibular surface mesh	-
`s_i`	Position of soft-tissue vertex i on the pre-operative surface	mm
`b_pre,j`	Position of mandibular vertex j on the pre-operative surface	mm
`b_post,j`	Position of mandibular vertex j on the augmented surface	mm
`delta_j`	Hard-tissue displacement of mandibular vertex j (b_post,j - b_pre,j)	mm
`N_i`	Index set of the K mandibular vertices nearest to s_i	-
`d_i,k`	Euclidean distance from s_i to the k-th nearest mandibular vertex	mm
`d_i,1`	Distance from s_i to its nearest mandibular vertex (local tissue depth)	mm
`sigma`	Gaussian bandwidth, derived from the data as sigma = c * d_bar	mm
`w_i,k`	Normalised Gaussian weight of the k-th neighbour of s_i	-
`M_i`	Deformation tensor at soft-tissue vertex i	-
`a_i`	Depth-based attenuation factor at soft-tissue vertex i	-
`u_i`	Predicted displacement of soft-tissue vertex i	mm

2. Parameters

Symbol	Value	Description
`K`	300	Number of nearest mandibular vertices contributing to each soft-tissue vertex. Interpretable only in relation to mandibular mesh density (see Implementation notes).
`c`	2	Dimensionless bandwidth scale factor. The Gaussian bandwidth is set globally as sigma = c * d_bar, where d_bar is the mean of d_i,k over all soft-tissue vertices and all K neighbours. In the present datasets d_bar was approximately 60 mm, giving sigma approximately 120 mm.
`rho`	0.3	Dimensionless scale factor of the depth-based attenuation term.
`n_iter`	30	Number of Laplacian smoothing iterations applied to the predicted surface.
`lambda`	0.05	Relaxation factor of the Laplacian smoothing operator.

3. Governing equations

Eq. 1   w_i,k = exp(-(d_i,k)^2 / (2 sigma^2)) / sum_k exp(-(d_i,k)^2 / (2 sigma^2)),   with sigma = c * d_bar

Eq. 2   M_i = I (3x3)

Eq. 3   a_i = exp(-d_i,1 / (rho * d_max)),   d_max = max_i d_i,1

Eq. 4   u_i = a_i * sum_{k=1..K} w_i,k * M_i * delta_{N_i,k}

Because the deformation tensor is initialised as the identity matrix, representing homogeneous and isotropic elastic behaviour, Eq. 4 reduces to a depth-attenuated, Gaussian-weighted propagation of the hard-tissue boundary condition. This simplification is deliberate and is what permits prediction in real time.

4. Pseudocode

```
INPUT
  B_pre    pre-operative mandibular surface mesh (native bone, including any
           pre-existing alloplastic implant)
  B_post   augmented mandibular surface mesh (native bone + designed PEEK
           implant), in vertex correspondence with B_pre
  S_pre    pre-operative soft-tissue surface mesh

PARAMETERS
  K = 300, sigma = 2, rho = 0.3, n_iter = 30, lambda = 0.05

OUTPUT
  S_pred   predicted post-operative soft-tissue surface mesh

----------------------------------------------------------------------

# Step 1: hard-tissue displacement field (boundary condition)
for j = 1 to M:
    delta_j <- b_post,j - b_pre,j

# Step 2: spatial query
T <- KDTree(B_pre)
for i = 1 to N:
    (N_i, d_i,1..K) <- T.query(s_i, K)      # ascending distance order

# Step 3: global Gaussian bandwidth
d_bar <- mean of d_i,k over all i = 1..N and all k = 1..K
sigma <- c * d_bar

# Step 4: normalised Gaussian weights           [Eq. 1]
for i = 1 to N:
    for k = 1 to K:
        w_i,k <- exp( -(d_i,k)^2 / (2 * sigma^2) )
    w_i,k <- w_i,k / sum over k of w_i,k

# Step 5: deformation tensor                    [Eq. 2]
for i = 1 to N:
    M_i <- I(3x3)          # homogeneous, isotropic elastic behaviour

# Step 6: depth-based attenuation               [Eq. 3]
d_max <- max over i of d_i,1
for i = 1 to N:
    a_i <- exp( -d_i,1 / (rho * d_max) )

# Step 7: soft-tissue displacement              [Eq. 4]
for i = 1 to N:
    u_i <- a_i * sum over k of ( w_i,k * M_i * delta_{N_i,k} )
    s_pred,i <- s_i + u_i

# Step 8: surface regularisation
S_pred <- LaplacianSmooth(S_pred, n_iter, lambda)

return S_pred
```

5. Implementation notes

Vertex correspondence. Steps 1 and 3 require that B_pre and B_post share a common topology. This is established by non-rigid registration of a template mandibular mesh to both the pre-operative and the augmented mandibular surface, as described in Section 2.5 of the main manuscript.

Mesh resolution. The parameter K is a vertex count and is therefore meaningful only relative to mandibular mesh density. In the present study the mandibular template mesh comprised approximately 17.000 vertices with a mean edge length of approximately 60 mm, and the resampled soft-tissue surface comprised approximately 30.000 vertices.

Bandwidth. Both sigma and d_max are computed globally from the input meshes rather than fixed a priori. Both therefore depend on the spatial extent of the soft-tissue surface supplied to the model. An identical cropping convention was applied to all cases.

Software. Implemented in Python 3.14.4 using NumPy 2.5.0, SciPy 1.18.0 (cKDTree for the nearest-neighbour search), trimesh 4.12.2 for mesh input and output, and PyVista 0.46.4 for Laplacian smoothing and surface handling.

Computational cost. The dominant terms are the k-d tree construction, O(M log M), and the K-nearest-neighbour query, O(N K log M).
